# NextGen sequence analysis of two sex-linked *P. tremuloides* genomic regions on chromosome 19

**DOI:** 10.1186/1753-6561-5-S7-P25

**Published:** 2011-09-13

**Authors:** Matthias Fladung, Birgit Kersten

**Affiliations:** 1 Johann Heinrich von Thünen Institute (vTI), Institute for Forest Genetics, Sieker Landstr. 2, D-22927 Grosshansdorf, Germany

## Background

Sex determination in poplars is still an open question. In the male *P. tremuloides* parent we identified several sex-linked SSR markers and mapped them to a central position on the male map of linkage group XIX.

## Material and methods

Using DNA probes derived from these SSR markers, two BAC clones were isolated from a BAC library of the *P. tremuloides* male clone (Pakull B. *et al.*, 2011, Can. J. For. Res. 41, 245-253) and further analyzed by454 sequencing (GATC Biotech AG). The generated single end reads were assembled to contigs using Newbler and Mira. The set of combined contigs (Newbler and Mira) of each BAC was subjected to scaffolding by SeqMan Pro (DNASTAR Lasergene) together with the related BAC end sequences created by Sanger sequencing (Pakull B. *et al.*, 2011, Can. J. For. Res. 41, 245-253). The subsequent combination of the created scaffolds to BAC consensus sequences was assisted by Sanger sequencing of PCR amplified scaffold ends and connections.

## Results

Mira created more contigs for both BACs than Newbler, where the N50-contig size was similar for both methods (~10,000 bp). The largest contig (38,518 bp) was assembled by Mira. Based on our strategy we already created a draft sequence of ~47,500 bp for one BAC clone. We expect a size of about 50 kb for the second BAC after finishing scaffolding.

## Conclusions

Based on the final consensus sequences of both BACs, we will search for putative sex-determining regions or genes in the *P. tremuloides* genome and compare them with *P. trichocarpa* paralogs.

